# G-Quadruplex Aptamer-Ligand Characterization

**DOI:** 10.3390/molecules27206781

**Published:** 2022-10-11

**Authors:** David Moreira, Daniela Leitão, Jéssica Lopes-Nunes, Tiago Santos, Joana Figueiredo, André Miranda, Daniela Alexandre, Cândida Tomaz, Jean-Louis Mergny, Carla Cruz

**Affiliations:** 1 CICS-UBI-Centro de Investigação em Ciências da Saúde, Universidade da Beira Interior, Av. Infante D. Henrique, 6201-506 Covilhã, Portugal; 2 Departamento de Química, Universidade da Beira Interior, Rua Marquês de Ávila e Bolama, 6201-001 Covilhã, Portugal; 3 Laboratoire d’Optique et Biosciences, Ecole Polytechnique, CNRS, INSERM, Institut Polytechnique de Paris, 91128 Palaiseau, France; 4 Institute of Biophysics of the CAS, v.v.i., Královopolská 135, 612 65 Brno, Czech Republic

**Keywords:** G-quadruplex aptamer, ligands, aptamer–ligand interactions, biophysical techniques

## Abstract

In this work we explore the structure of a G-rich DNA aptamer termed AT11-L2 (TGGTGGTGGTTGTTGTTGGTGGTGGTGGT; derivative of AT11) by evaluating the formation and stability of G-quadruplex (G4) conformation under different experimental conditions such as KCl concentration, temperature, and upon binding with a variety of G4 ligands (360A, BRACO-19, PDS, PhenDC3, TMPyP4). We also determined whether nucleolin (NCL) can be a target of AT11-L2 G4. Firstly, we assessed by circular dichroism, UV and NMR spectroscopies the formation of G4 by AT11-L2. We observed that, for KCl concentrations of 65 mM or less, AT11-L2 adopts hybrid or multiple topologies. In contrast, a parallel topology predominates for buffer containing 100 mM of KCl. The *T*_m_ of AT11-L2 in 100 mM of KCl is 38.9 °C, proving the weak stability of this sequence. We also found that upon titration with two molar equivalents of 360A, BRACO-19 and PhenDC3, the G4 is strongly stabilized and its topology is maintained, while the addition of 3.5 molar equivalents of TMPyP4 promotes the disruption of G4. The *K*_D_ values between AT11-L2 G4, ligands and NCL were obtained by fluorescence titrations and are in the range of µM for ligand complexes and nM when adding NCL. In silico studies suggest that four ligands bind to the AT11-L2 G4 structure by stacking interactions, while the RBD1,2 domains of NCL interact preferentially with the thymines of AT11-L2 G4. Finally, AT11-L2 G4 co-localized with NCL in NCL-positive tongue squamous cell carcinoma cell line.

## 1. Introduction

Aptamers are short, single-stranded DNA or RNA sequences that are capable of selectively interacting with their target molecules due to an appropriate conformation [1]. Aptamers have been considered for a wide range of biomedical applications, namely in clinical diagnostics and as therapeutic agents [2]. Compared to other classes of ligands such as monoclonal antibodies or peptides, aptamers have several advantages, such as small size, good biocompatibility, stability, lack of immunogenicity, easy enzymatic or chemical synthesis and modification, rapid tissue penetration, and cell internalization [3,4,5]. Consequently, they can be used in different delivery systems with direct therapeutic potential in the circulatory system or in cells, such as drug carriers to facilitate specific cellular recognition and uptake [6].

Some aptamers sequences, rich in guanines, can form G-quadruplexes (G4), which are organized by the consecutive stacking of two or more G-quartets [7]. One example is AT11, derived from AS1411, a G4-forming aptamer that reached clinical trials. AT11 forms a single major G4 conformation comprising two propeller-type parallel-stranded subunits that are connected through a central linker [8]. The target of AT11 aptamer is nucleolin (NCL), a protein involved in the regulation of several mechanisms related to nucleic acid metabolism and whose expression is correlated with increased cancer aggressiveness [9].

In 2017, Phan et al., identified more derivatives of AT11 aptamer, termed AT11-B1, AT11-B0, AT11-L0 and AT11-L2 [8]. Recently, our group showed that AT11, AT11-B0, AT11-L0 and AT11-B1 recognized NCL, and these aptamers can be used as potential drug delivery systems for cervical and tongue cancer cell lines [10,11,12,13]. Moreover, we also found that improving G4 aptamer stability through changing linker/bulge/loop nucleotides at the appropriate positions and associated ligands could also be a strategy to select a preferential conformation and obtain a more selective drug delivery system [10,11,12,13].

Hence, in this study, we characterized a derivative of AT11, termed AT11-L2, in which one thymine was added to the linker, and analyzed the change of structure upon ligand and NCL binding. For this purpose, we evaluated the formation and stabilization of the G4-forming motif in AT11-L2 using circular dichroism, UV and NMR spectroscopy, fluorescence titrations and molecular modeling studies. Moreover, the subcellular localization of the AT11-L2/PhenDC3 complex with NCL was assessed by fluorescence confocal microscopy in UPCI-SCC-154 and NHDF cells.

## 2. Results and Discussion

**Figure 1:**
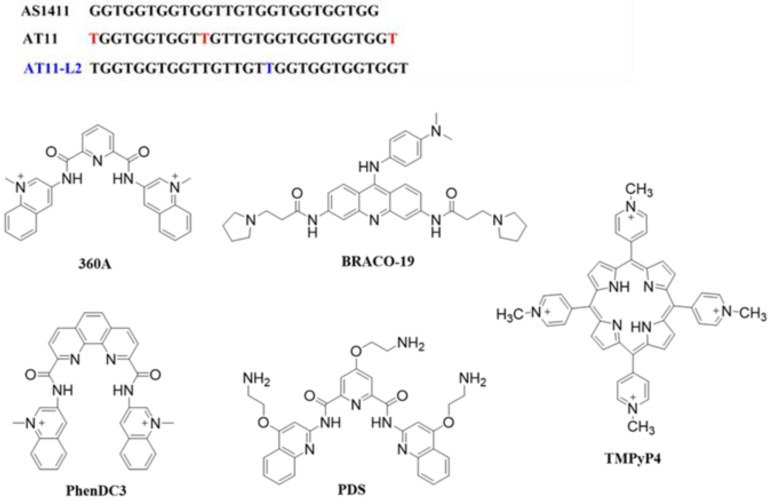
AS1411 derivatives and chemical structure of G4 ligands.

Nucleic acid aptamers constitute an interesting class of pharmaceuticals [14] and, due to their ability to bind with high specificity to target molecules, have been explored as systems for cancer cell targeting [15]. In this sense, aptamers containing G4-forming motifs are particularly interesting and can recognize and selectively bind the protein NCL [16]. AT11 is an AS1411 aptamer derivative that can form a major G4 conformation and have similar antiproliferative activities [8]. Additionally, AT11 derivatives were designed to study the influence between bulge/linker size on the thermal stability of AT11 [8]. The previous results indicate that a decrease in either the bulge or linker size of AT11 leads to an increase in thermal stability (AT11-B1, AT11-B0 and AT11-L0) [8]. The AT11 derivatives have also been studied as delivery systems for cervical and oral cancer cells [11,12,13]. However, the AT11-L2 derivative remains unexplored. Herein, we explore the AT11-L2 sequence and its interaction with well-known G4 ligands 360A, BRACO-19, PhenDC3, TMPyP4 and PDS (Figure 1). Additionally, we evaluated if AT11-L2 and AT11-L2/ligand complexes can recognize NCL.

**Figure 2:**
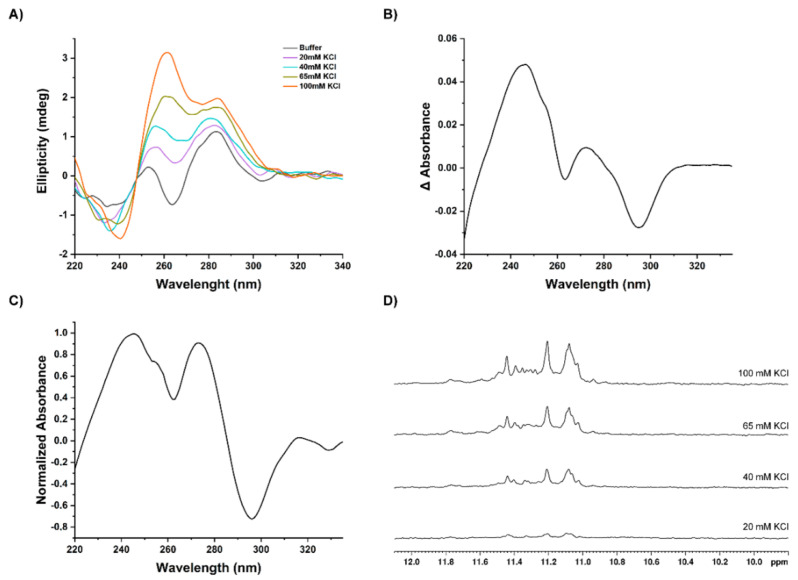
Evaluation of G4 formation using distinct spectroscopic techniques. (**A**) CD spectra of AT11-L2 (10 μΜ) in 10 mM of lithium cacodylate buffer with increasing concentrations of KCl in the range of 220–340 nm. (**B**) IDS spectrum resultant from the difference of UV spectra in the presence and absence of KCl. (**C**) TDS spectrum of AT11-L2 in lithium cacodylate containing 100 mM of KCl, obtained by subtracting the spectrum in folded and unfolded states and then normalized relative to the maximum absorbance (**D**) Effect of KCl salt on the structure formation of AT11-L2 G4 (50 μΜ) was monitored by ^1^H NMR spectroscopy recorded in lithium cacodylate supplemented with 10% D_2_O and increased concentrations of KCl (20 to 100 mM). All measurements were performed at 20 °C.

First, we evaluated the ability of AT11-L2 to fold into a G4 by the addition of KCl directly to the cuvette using CD spectroscopy. The spectra are depicted in Figure 2A. The results show that only in the presence of 65 mM of KCl does AT11-L2 fold into a hybrid G4 topology, as evidenced by the characteristic bands around 260 nm and 290 nm [17]. In addition, we observe that increasing concentrations of KCl result in an increase in the ellipticity around 260 nm, indicating a predominant parallel topology of AT11-L2. Then using UV-Vis spectroscopy, we performed IDS and TDS measurements (Figure 2B,C) [18]. The IDS experiments were performed to elucidate the G4 formation under an isothermal process. The IDS spectrum (Figure 2B) was obtained from the difference between the folded (presence of KCl) and unfolded state (absence of KCl). The formation of G4 is proved by the hypochromism at 260 nm and 290 nm.

In the TDS experiment, the temperature was variable in the process and recorded a spectrum in a folded and unfolded state, i.e., above and under the melting temperature (*T*_m_). The achieved spectrum is presented in Figure 2C and again denotes the ability to fold in G4, exhibiting characteristic peaks at 245 nm, 273 nm and 295 nm [19]. The hypochromic band at 295 nm can be explained by the tetrads stacking in the folding (n → π * and π → π * transitions) and is typical of the G4 topologies [19,20].

We confirmed these results by ^1^H NMR spectra and repeated the same titration points. For this, 1 M of KCl solution was directly added to the 3 mm NMR tube. The ^1^H NMR spectra (Figure 2D) showed the imino proton resonances at 11–11.5 ppm, which are characteristic of Hoogsteen hydrogen bonds, upon the addition of 40 mM of KCl. The spectra suggest a single G4 conformation, and the signals with low intensity can be indicative of minor G4 conformations. Additionally, to access the temperature effect on the G4 structure and stability, it was increased to 37 °C under the same ionic conditions (100 mM of KCl) (Figure S1). The spectrum presented in Figure S1 showed the loss of all imino signals characteristic of G4, indicating weak thermal stability of the G4 in AT11-L2. This result is in accordance with that previously reported by Phan and collaborators on a related sequence [8].

**Figure 3:**
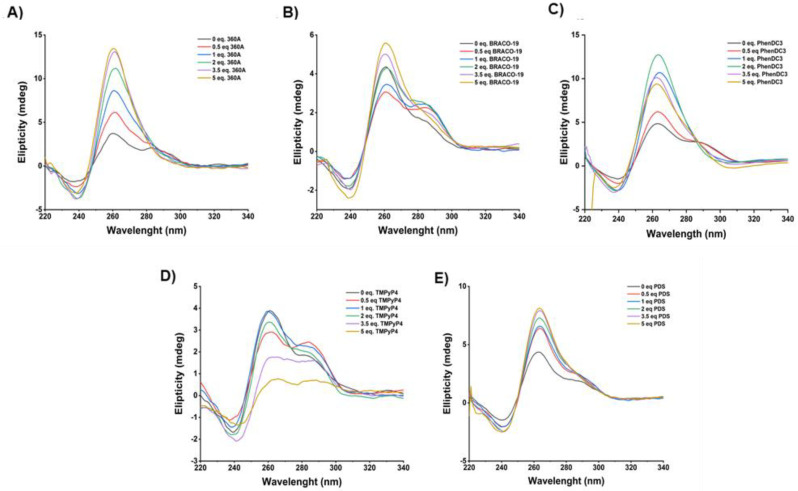
CD spectra of AT11-L2 in the absence and presence of increased molar equivalents of ligands (**A**) 360 A, (**B**) BRACO-19, (**C**) PhenDC3, (**D**) TMPyP4 and (**E**) PDS. CD spectra were acquired in a buffer containing 10 mM of lithium cacodylate and 100 mM of KCl.

To maintain the G4 structure under physiological conditions we added G4 ligands to a pre-folded AT11-L2 G4 solution, and we determined the topology (Figure 3) and melting temperature (Table 1) by CD spectroscopy. Upon the addition of five molar equivalents of 360A, BRACO-19 and PDS (Figure 3A,B), we observed an increase in ellipticity and a parallel topology with the disappearance of band at 290 nm, and the appearance of a positive band at 260 nm and a negative one at 240 nm. In addition, PhenDC3 promoted the formation of a parallel topology with an increase in ellipticity until the addition of two molar equivalents. Upon the addition of three and five molar equivalents of PhenDC3, a decrease in ellipticity was observed; however, the signature of typic parallel topology was maintained.

These results suggest that 360A, BRACO-19, PhenDC3 and PDS can stabilize and change the topology of pre-folded AT11-L2 G4 from hybrid to parallel. Conversely, upon the titration of two molar equivalents of TMPyP4 (Figure 3D), no changes in pre-folded G4 hybrid topology were observed. Further, with the addition of 3.5 molar equivalents of TMPyP4, a very important decrease in ellipticity was observed, indicating that TMPyP4 promoted the unfolding of the AT11-L2 quadruplex under these conditions.

**Figure 4:**
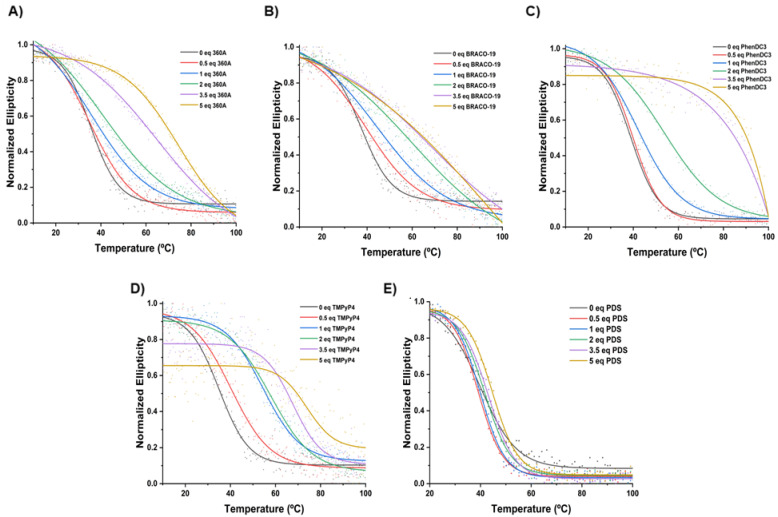
CD melting spectra of AT11-L2 in the absence and presence of increased molar equivalents of ligands (**A**) 360A, (**B**) BRACO-19, (**C**) PhenDC3, (**D**) TMPyP4 and (**E**) PDS. CD spectra were acquired in a buffer containing 10 mM of lithium cacodylate (pH 7.2) and 100 mM of KCl.

**Figure 5:**
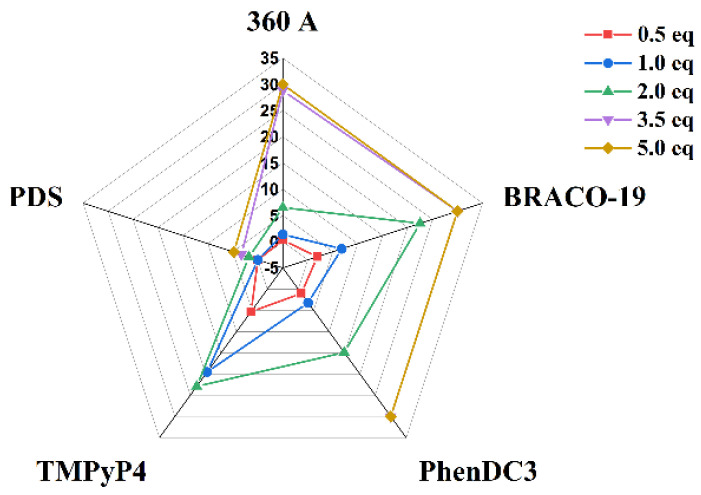
*T*_m_ radar plot of AT11-L2 in the presence of different molar equivalents of tested ligands obtained by CD-melting experiments.

The melting temperature (*T*_m_) of AT11-L2 G4 was determined in the absence and presence of 360A, BRACO-19, PhenDC3 and TMPyP4 (Figure 4 and Figure 5 and Table 1) from the wavelength of maximum CD ellipticity (262 nm). The *T*_m_ value of AT11-L2 G4 in the presence of 100 mM of KCl is only 38.9 °C, barely above physiological temperature, in agreement with weak thermal stability [8]. Upon the addition of different concentrations (5 to 50 µM) of the tested ligands, except PDS, we observed an increase in the *T*_m_ values, which indicates ligand-induced stabilization of the AT11-L2 G4 in a concentration-dependent manner. PDS started to stabilize the AT11-L2 sequence only after adding two molar equivalents, inducing a low thermal stabilization when compared with other tested ligands. TMPyP4 is the most stabilizing ligand (Δ*T*_m_ = 22.9 °C) at 2 molar equivalents, followed by BRACO-19 (Δ*T*_m_ = 22.5 °C), PhenDC3 (Δ*T*_m_ = 14.9 °C), 360A (Δ*T*_m_ = 6.5 °C) and PDS (Δ*T*_m_ = 1.8 °C).

Upon the addition of 3.5 molar equivalents, all ligands except 360A and PDS promoted a stabilization over 30 °C for AT11-L2 G4. However, the *T*_m_ results with TMPyP4 titration may not be a result of the thermal stabilization of the G4 structure but due to the ligand-induced conformational forms, as represented in Figure 3D. Our results can indicate that TMPyP4 destabilizes the G4 in a dose-dependent manner, and this profile has already been reported by other groups [21,22,23]. In this sense, the obtained Δ*T*_m_ values after adding 3.5 molar equivalents of TMPyP4 may not be a result of the thermal stabilization of the G4 structure, but a stabilization of a single strand. Indeed, even when a single binding site is high affinity on a G4, multiple binding sites in the single strand will prevail if the ligand is in excess.

**Figure 6:**
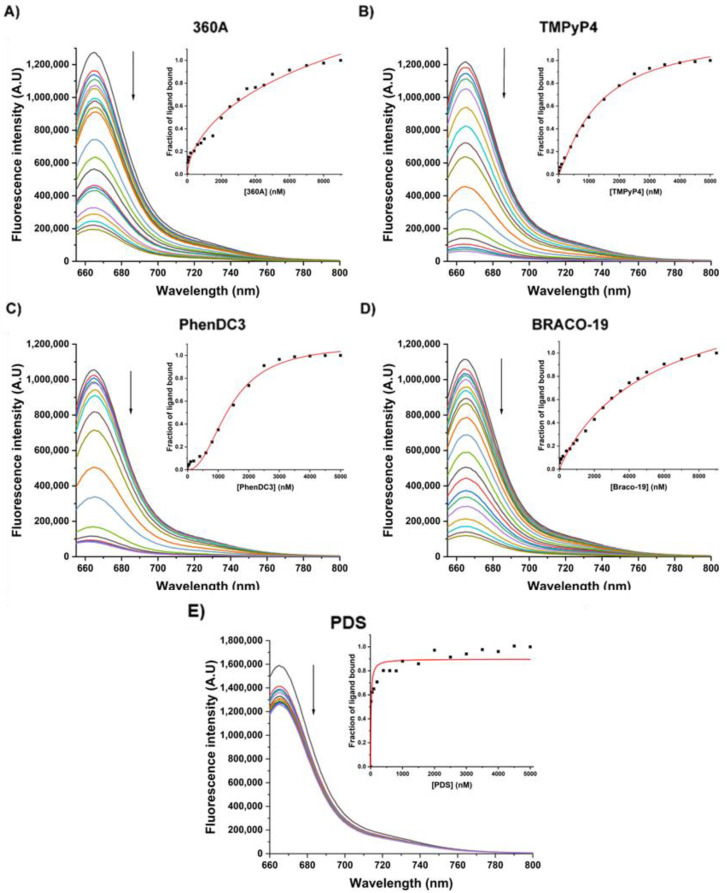
Fluorescence titration spectra of pre-folded 5′-Cy5-AT11-L2 G4 with increasing concentrations of (**A**) 360A, (**B**) TMPyP4, (**C**) PhenDC3, (**D**) BRACO-19 and (**E**) PDS. The experiments were performed in buffer containing 10 mM of lithium cacodylate and 100 mM of KCl with excitation set at 647 nm, and emission was recorded ranging from 655 to 800 nm.

We further determined the apparent equilibrium constants (*K*_D_) of these ligands via fluorometric titrations (Figure 6). The experiments were performed by titrating the ligands directly to the pre-folded labeled 5′-Cy5-AT11-L2 G4. Upon adding the ligands, a decrease in emission intensity was observed. The results from the fluorescence titrations were fitted using Hill saturation binding mode and obtained *K*_D_ values of 3.1 × 10^−5^ M, 1.3 × 10^−6^ M, 1.4 × 10^−6^ M, 5.6 × 10^−6^ M for 360A, TMPyP4, PhenDC3 and BRACO-19, respectively (Table 2). The results for PDS titration experiments were fitted using Michaelis–Menten mode and obtained a *K*_D_ value of 1.4 × 10^−8^ M. The *K*_D_ values in the range 10^−5^ and 10^−8^ suggest weak-to-moderate complex formation, which agrees with the ligand-induced stabilization obtained in CD melting experiments. Overall, PDS (*K*_D_ = 1.4 × 10^−8^ M), TMPyP4 (*K*_D_ = 1.3 × 10^−6^ M) and PhenDC3 (*K*_D_ = 1.4 × 10^−6^ M) presented the highest affinity and acted in a cooperative binding mode, as suggested by the Hill coefficient (*n*) above 1 (Table 2).

The complex AT11-L2 G4/PhenDC3 was further investigated by ^1^H NMR at 20 °C and 37 °C. PhenDC3 was chosen because it induced a moderate *T*_m_ increase at two molar equivalents, maintaining the parallel topology of AT11-L2 G4, and it has a *K*_D_ in the micromolar range.

Titrations performed with two molar equivalents of PhenDC3 (Figure S2) resulted in a broadening of the imino protons, suggesting a poorly defined binding mode of the ligand to the G4 structure and/or the formation of higher molecularity complexes [13].

In this experiment, DMSO-*d*_6_ was added until it reached 8% of the final volume, in order to avoid PhenDC3 aggregation, as previously described [13]. Additionally, the NMR spectrum was acquired to check if the G4 structure was maintained under 37 °C in the presence of PhenDC3. The spectra presented in Figure S3 evidenced that G4 is maintained in the presence of PhenDC3 by the appearance of the imino protons in the 10–12 ppm range, which is characteristic of Hoogsteen base pairs.

Since AT11-L2 is an AT11 derivative (itself a modified version of AS1411) reported as NCL aptamers [8,11,13], it was of interest to investigate the binding affinity and ligands complex formation of AT11-L2 G4 with NCL. The *K*_D_ values of 5′-Cy5-AT11-L2 G4/ligand complex towards NCL were determined by fluorometric titrations and fitted to Hill saturation binding mode or Michaelis–Menten (Figure S4). The *K*_D_ values are 6.4 × 10^−6^, 1.5 × 10^−7^, 1.8 × 10^−8^, 1.2 × 10^−9^ and 6.3 × 10^−8^ for complexes formed by 5′-Cy5-AT11-L2 and 360A, BRACO-19, PhenDC3, TMPyP4 and PDS, respectively, with NCL.

After that, restrain-assisted molecular docking and molecular dynamics simulations were performed in order to understand how AT11-L2 G4 interacts with the ligands 360A, BRACO-19, PhenDC3, TMPyP4, PDS and NCL.

Since no high-resolution solution structure of AT11-L2 is currently available, while the 3D AT11 structure was already determined by NMR spectroscopy [8], we modified the sequence of AT11 and optimized it by running a 200 ns fully solvated MD run. Figure S5 shows the final snapshots of the AT11-L2 structure (top and side views), and the RMSF and RMSD plots. RMSF demonstrated that residues mostly affected during the entire run are mainly thymines that belong to the linker and the bulge or the neighboring guanines. On the other hand, the guanines constituting the G-tetrads showed minor fluctuations. RMSD showed that the AT11-L2 G4 structure did not significantly change the position along the 200 ns MD run. Then, the final frame of the AT11-L2 200 ns MD simulation was used as a scaffold for docking experiments with ligands and NCL. Following the docking experiments, the best conformers in terms of binding energy, previously referred to, were further examined through MD simulations.

**Figure 7:**
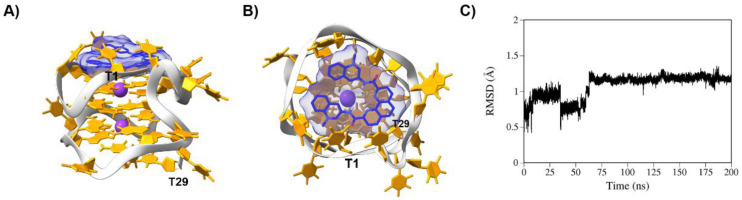
Final snapshots of 200 ns MD simulations of the complex between AT11-L2 G4 and PhenDC3. (**A**) Side view and (**B**) top view. The backbone is highlighted in light grey, while nucleotides are depicted in orange. K^+^ is shown in purple. The ligand is depicted in blue. The first and last nucleotides of AT11-L2 are also shown. (**C**) RMSD plot of the 200 ns simulation of AT11-L2 G4/PhenDC3 complex.

The four ligands mainly bind to the AT11-L2 G4 structure by stacking interactions. The planar nature of the aromatic core of the ligands seems to favor that type of interaction with the G4 structure. Specifically, PhenDC3 binds to a cavity between the top G-tetrad (G2-G5-G8-G12) and nucleobases T1, T4 and T14, which build up a cap over the ligand (Figure 7). Despite an initial RMSD fluctuation of PhenDC3 on the G4 structure in the first 75 ns of MD simulation, the complex achieved stability throughout the remaining simulation time. BRACO-19 interacts with the AT11-L2 G4 structure via stacking interactions with nucleobases T7 and T11 (Figure S6A). 360A binds to nucleobases G5, T11 and T14 (Figure S6B). PDS binds via stacking interactions to a cavity between nucleobases T1 and T11 on the top, and T4, T7 and T14 on the bottom. Moreover, PDS established hydrogen bonds with T11 and T14 (Figure S6C). Finally, TMPyP4 binds to the top of the AT11-L2 G4 structure, specifically to nucleobases T11 and T14 (Figure S6D).

However, the experimental results indicate that TMPyP4 destabilizes the AT11-L2 G4 structure. The most probable hypothesis is that the ligand binds through stacking interactions to the top G-tetrad and initiates the destabilization of the G4 structure [21]. Notwithstanding, the binding mode of TMPyP4 to G4s remains ambiguous. Recently, some reports described that it destabilizes/unfolds G4s [21,24,25], while the first ones reported the binding and stabilization of the G4 structures by TMPyP4 [26].

Overall, the results of in silico studies predicted that, although they have the same binding mode, the ligands can depict a different effect on the stability of the G4 structure, as previously observed by biophysical experimental results.

**Figure 8:**
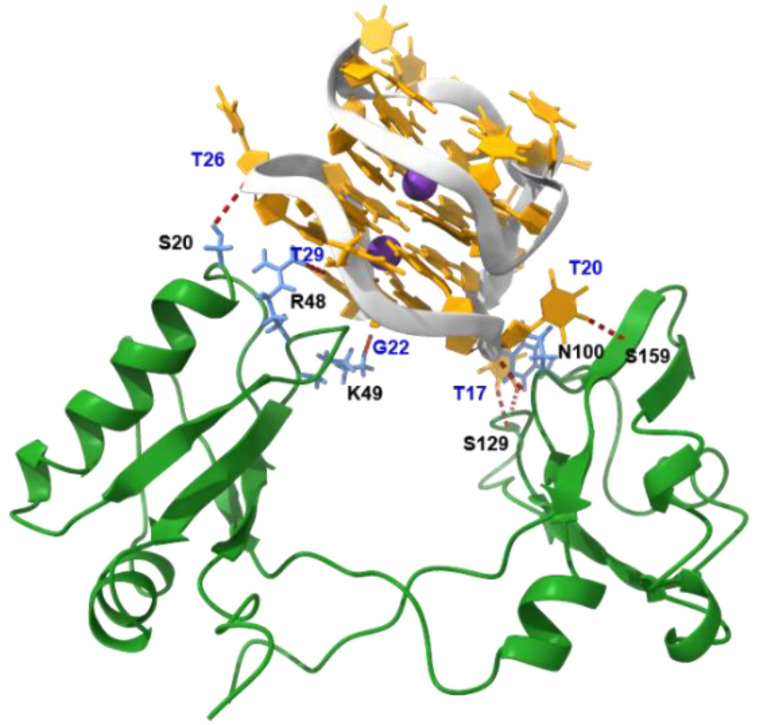
Final snapshot of 200 ns MD simulations of the complex AT11-L2 G4/NCL RBD1,2. G4 backbone is depicted in light grey, while nucleotides are highlighted in orange. K^+^ is shown in purple. NCL residues that interact with AT11-L2 G4 by hydrogen bonding are highlighted in cornflower blue.

The binding of AT11-L2 G4 to NCL was also evaluated by MD simulations. After an MD run of 200 ns, the AT11-L2 G4 structure moves to the cavity formed between RBD1,2, and developed almost the same interactions with both RBDs (Figure 8). We previously observed this type of binding mode for the AT11-B0 G4 aptamer [11]. On the other hand, another aptamer derivative termed AT11-B1 seems to mainly interact with RBD2 [13]. Furthermore, a total of nine H-bonds (red dashed lines) is established between the AT11-L2 G4 structure and NCL. Thymines T17, T20, T26 and T29 interact with RBD1,2, thus, demonstrating the preference of NCL for the loop residues. It is worth noting that these nucleobases are located on the opposite side where the ligands are expected to bind, which probably means that ligands do not interfere with the binding of the AT11-L2 G4 structure to NCL.

**Figure 9:**
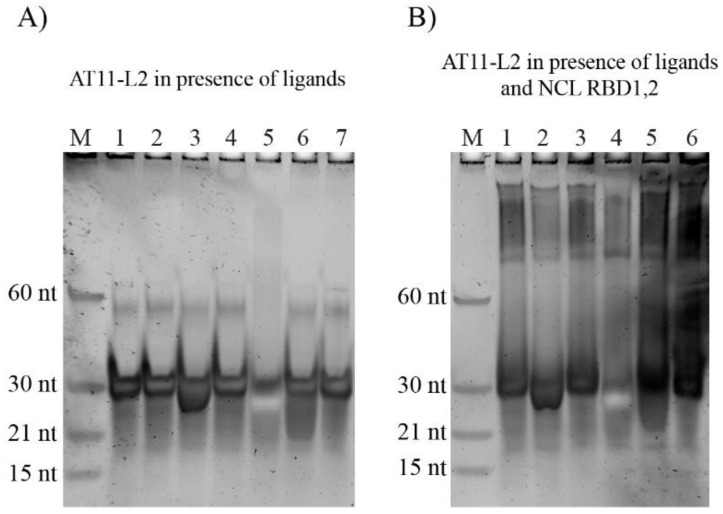
Non-denaturing gel electrophoresis of AT11-L2 G4 (2 μM) in (**A**) presence of ligands (4 μM) (Lanes: M—Marker; 1—ultrapure water; 2—annealing buffer; 3—360A; 4—PhenDC3; 5—TMPyP4; 6—BRACO-19 and 7—PDS) and (**B**) Ligands (4 μM) and NCL (2 μM) (Lanes: M—Marker; 1–NCL; 2—360A; 3—PhenDC3; 4—TMPyP4; 5—BRACO-19 and 6-PDS).

The molecularity of the AT11-L2 G4 complex with ligands and NCL was evaluated by PAGE experiments. Ligands and NCL were added to the pre-annealed non-labelled AT11-L2 sequence in a proportion of 1:2 and 1:1, respectively. The electrophoretic profiles are presented in Figure 9 and indicate that AT11-L2 G4 migrates predominantly as a monomer (around 29 nt). These data agree with the CD and NMR experiments. The profile also shows a less intense band below 60 nt, suggesting the formation of a small fraction of G4 dimer around 58 nt. The profile is similar in both water and in annealing buffer.

The complexes with PhenDC3, BRACO-19 and PDS display the same major conformation when compared with the AT11-L2 G4. For 360A, it displays a smear band below the main band, suggesting the formation of other molecular species that are not structurally defined. Finally, TMPyP4 shows a less intense band, which probably indicates ligand-induced conformational forms, as shown in CD.

Next, PAGE experiments were performed to evaluate the AT11-L2 G4/ligand/NCL complexes. These experiments were performed in order to evaluate whether the ligands changed the electrophoretic profile of NCL towards AT11-L2. None of the tested ligands impaired protein recognition by AT11-L2 G4.

**Figure 10:**
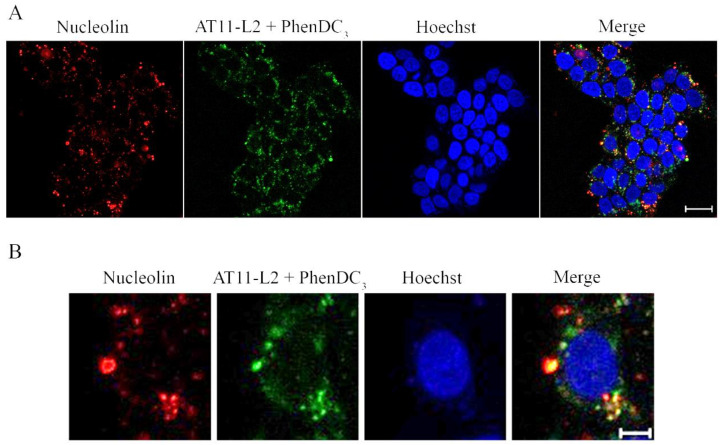
NCL immunofluorescence in UPCI-SCC-154 cells with 5´-FAM-AT11-L2-3´-TAMRA (green) conjugate with PhenDC3. The anti-NCL primary antibody was conjugated with an Alexa Fluor 647 secondary antibody (red) and nuclei were stained with Hoechst (blue). (**A**) Original images obtained with a 63× objective (scale bar: 20 µm). (**B**) Magnification from the original image (scale bar: 5 µm).

Finally, the subcellular localization of the AT11-L2/PhenDC3 complex with NCL in UPCI-SCC-154 and NHDF cells was evaluated by fluorescence confocal microscopy. The UPCI-SCC-154 tongue oral squamous carcinoma cell line was used, since we have previously shown by Western blot and confocal microscopy that NCL is overexpressed in these cells when compared to NHDF (normal human dermal fibroblasts, NHDF) [13]. We followed the intrinsic fluorescence of 3′-TAMRA of labeled AT11-L2, while the primary anti-NCL antibody conjugated with the secondary antibody AlexaFluor 647^®^ allowed the localization of NCL at the cell surface. As seen in Figure 10 and Table S1, complex 5’-FAM-AT11-L2-3´-TAMRA/PhenDC3 is able to localize NCL in UPCI-SCC-154 cells. The co-localization of the complex and NCL was determined by measuring the Manders’ coefficients M1 and M2 (Table S1) [27]. These coefficients range from 0 to 1 and indicate the fraction of intensity in a channel that is located in pixels where there is above-zero intensity in the other color channel. In the obtained images, the average fraction of 5´-FAM-AT11-L2-3´-TAMRA/PhenDC3 that colocalized with the imaged NCL (M2 coefficient presented in Table S1) was 0.733 ± 0.030, suggesting that a high proportion of the complex was able to colocalize with the studied protein.

The obtained results are in accordance with previous studies in which AS1411 derivatives were able to colocalize with NCL that was expressed at the cell surface of cancer cells (cervical and oral cancer cells), even when bound to G4 ligands (namely, acridine orange derivatives and PhenDC3) [10,11,13].

## 3. Materials and Methods

### 3.1. Oligonucleotides and Ligands

The oligonucleotide sequence AT11-L2 (5′-TGGTGGTGGTTGTTGTTGGTGGTGGTGGT-3′), was purchased from Eurofins Genomics (Germany) with HPLC-grade purification. Labelled oligonucleotide sequences (5′-Cy5-AT11-L2 and 5′-FAM-AT11-L2-TAMRA-3′) were acquired from Eurogentec (Belgium). All sequences were used without further purification. Stock solutions were prepared using Milli-Q water and stored at −20 °C until being used. The oligonucleotide sample concentration was determined on a UV–Vis spectrophotometer (Thermo Scientific™ Evolution 220, Saint Louis, MO, USA) by measuring the absorbance at 260 nm and using the molar extinction coefficient (ε) of 272,900 and 305,200 L∙mol^−1^∙cm^−1^ for unlabeled and labeled sequences, respectively. Before each experiment, the oligonucleotide was annealed in 10 mM of lithium cacodylate (pH 7.2) that was supplemented with 100 mM of KCl (annealing buffer) by heating for 10 min at 95 °C, followed by a cooldown of 10 min on ice.

Ligands 360A (2,6-N,N′-methyl-quinolinio-3-yl)-pyridine dicarboxamide); CAS: 794458-56-3), BRACO-19 (N,N′-(9-(4-(dimethylamino)phenylamino)acridine-3,6-diyl)bis(3-(pyrrolidin-1-yl)propanamide); CAS: 1177798-88-7), PhenDC3 (3,3′-[1,10-phenanthroline-2,9-diylbis(carbonylimino)]bis [1-methylquinolinium] 1,1,1-trifluoromethanesulfonate; CAS: 929895-45-4); and TMPyP4 (tetra-(N-methyl-4-pyridyl)porphyrin; CAS: 36951-72-1) were acquired from Sigma-Aldrich (Waltham, MA, USA). PDS (Pyridostatin; CAS: 1085412-37-8) was acquired from ChemMedExpress (Monmouth Junction, NJ, USA). The ligands were stocked in a 10 mM dimethyl sulfoxide (DMSO; Thermo Fisher Scientific, USA) solution and further dilutions were carried out using Milli-Q water. The recombinant NCL RBD1,2 used in all experiments was synthesized and purified as reported previously [24,28].

### 3.2. Circular Dichroism

AT11-L2 was dissolved in 10 mM of lithium cacodylate (Sigma-Aldrich, USA) at pH 7.2 at a final concentration of 10 µM and annealed as previously described. A 1 mm path-length quartz cuvette (Hellma, Germany) was used with AT11-L2 at 10 µM in 10 mM lithium cacodylate buffer at a final volume of 300 µL.

The experiments started with the titration directly in a quartz cuvette by adding increased concentrations of KCl (20 to 100 mM). After each salt addition, the CD spectrum was acquired through wavelengths varying from 200 to 320 nm, with a scan speed of 100 nm/min, 1 nm bandwidth and 1 s integration time over 3 averaged accumulations using a Jasco J-815 spectropolarimeter (Tokyo, Japan) equipped with a Peltier temperature controller (model CDF-426S/15).

For ligand titrations, the same protocol was employed, using an annealing buffer composed of 10 mM of lithium cacodylate (pH = 7.2) that was supplemented with 100 mM of KCl. The required volume for the ligand titrations was included in the cuvette containing pre-folded G4 AT11-L2.

For each ligand titration point, CD melting experiments were performed to determine the melting temperature (*T*_m_) under each condition. These experiments were performed in the temperature range of 10–100 °C, with a heating rate of 4 °C/min by monitoring the ellipticity at 260 nm.

The results were converted into fraction folded plots (θ), corresponding to Equation (1), which were adjusted to a Boltzmann distribution, using OriginPro2021.

(1) $$\theta =\frac{CD-CD_{260\;nm}^{\min}}{CD_{260\;\mathrm{nm}}^{\max}-CD_{260\;\mathrm{nm}}^{\min}}$$

The melting temperature (*T*_m_) was calculated through the two-state mid-transition point. The CD value is the molar ellipticity at 260 nm at each temperature and CD^min^ and CD^max^ are the lowest and highest molar ellipticities, respectively.

### 3.3. NMR Spectroscopy

For the KCl titration experiments, the unlabeled AT11-L2 sequence at a concentration of 50 µM was dissolved in 10 mM of lithium cacodylate (pH 7.2) containing 10% (*v*/*v*) D_2_O (Eurisotop, France) in a 3 mm tube with a total volume of 180 µL. After each salt addition, the sample was annealed in a 3 mm tube as previously described. The NMR spectra were acquired on a 600 MHz Bruker Avance III containing a 5 mm inverse detection z gradient QCI cryoprobe operating at a Larmor ^1^H frequency of 600.10 MHz at 293.15 K. The ^1^H NMR spectra were obtained using a water suppression pulse sequence (zgesgp according to the Bruker standard library), where a 2 s relaxation delay, 32 K data points and 1024 scans were used for a 12.019 Hz spectral width centered on the water resonance. Chemical shifts (δ) were measured in ppm. All spectra were analyzed using Bruker TopSpin4.1.

### 3.4. UV Spectroscopy

#### 3.4.1. Thermal Difference Spectroscopy (TDS)

For the TDS experiments, 6 µM of AT11-L2 was annealed in 10 mM of lithium cacodylate buffer (pH = 7.2) that was supplemented with 100 mM of KCl. The absorbance spectra were recorded on UV–vis spectrophotometer (Thermo Scientific™ Evolution 220, Waltham, MA, USA) using 1 cm path-length quartz cells (Hellma, Munich, Germany) with a scan range of 220–335 nm and a scan rate of 200 nm/min, as previously described [29].

The first spectrum was recorded at 20 °C and then the temperature was increased to 90 °C, at which point the second spectrum was recorded. The TDS spectrum was obtained by subtracting the absorbance spectrum at 20 °C (folded state) from the one at 90 °C (unfolded state). The data were normalized relative to the maximum absorbance.

#### 3.4.2. Isothermal Difference Spectra (IDS)

For IDS experiments, 6 µM of AT11-L2 was annealed in 10 mM of lithium cacodylate buffer (pH = 7.2). The first spectrum was recorded at 25 °C in the absence of KCl. Then, KCl was added directly to the quartz cuvette to reach a final concentration of 100 mM, and the spectrum was recorded after 15 min of equilibration. The IDS spectrum was obtained by subtracting the unfolded absorbance spectrum (absence of KCl) from the folded one (presence of KCl).

### 3.5. Fluorescence Spectroscopy

Fluorescence titrations were performed on a Horiba FluoroMax4 fluorometer (Tokyo, Japan), which was equipped with a Peltier-type temperature control system, using a quartz suprasil cuvette (10 × 4 mm) and a volume of 700 µL. All spectra were scanned with an integration time of 0.5 s, an emission and an excitation slit width of 1 nm and averaged over three scans. Cy5-AT11-L2 was excited with a wavelength of 647 nm. Titrations were performed by adding successive ligand or NCL stock solutions to the previously annealed Cy5-AT11-L2 (1 µM), followed by 10 min of equilibration time at each titration point.

Fluorescence data obtained were converted into a fraction of bound ligand (α) plots using Equation (2):

(2) $$\alpha =\frac{I-I_{\lambda }^{free}}{I_{\lambda }^{bound}-I_{\lambda }^{free}}$$

where *I* is the fluorescence intensity at 665 nm for each DNA:ligand ratio and *I^free^* and *I^bound^* are the fluorescence intensities of the free and fully bound DNA, respectively. Data points were then fitted into the Hill saturation binding model or Michaelis–Menten model using OriginPro 2021, and *K*_*D*_ values were determined from the following Equation (3):

(3) $$\alpha =\frac{{\left[DNA\right]}^{n}}{K_{D}+{\left[DNA\right]}^{n}}$$

In which *K*_*D*_ is the apparent equilibrium dissociation constant, [DNA] is the concentration of the DNA and *n* is the Hill constant which defines the cooperativity of ligand binding. For the experiments with PDS, data points were fitted to the Michaelis–Menten model.

(4) $$\alpha =\frac{\left[DNA\right]}{K_{D}+\left[DNA\right]}$$

### 3.6. Molecular Docking and Molecular Dynamics (MD) Simulations

Since the 3D structure of AT11-L2 is not available, a predicted model was built based on the AT11 structure (PDB: 2N3M) [8]. The structure was optimized for further experiments by running fully solvated 200 ns MD simulations using GROMACS 2021.4 with the following parameters. The final snapshot of the 200 ns simulation was applied as a scaffold for further experiments.

The AT11-L2, ligands (360A, BRACO-19, PDS, PhenDC3, TMPyP4) and NCL RNA-binding domains (RBD1,2–PDB: 2KRR) were optimized for docking using the Dock Prep tool of Chimera 1.15. The procedure for molecular docking has been previously described in detail [11]. The most representative structures were selected and further processed with MD simulations of the all-atom force field OL15.

For MD simulations in GROMACS 2021.4, the prominent conformers from molecular docking studies were used as scaffolds. The ACPYPE program produced the ligand’s topology and parameters files in GROMACS-compatible format. Explicit solvent MD runs of 200 ns were performed for AT11-L2/ligands and AT11-L2/NCL complexes. The procedure for MD studies has previously been described in detail [11]. All figures were rendered using UCSF ChimeraX 1.2.5.

### 3.7. Non-Denaturing Polyacrylamide Gel Electrophoresis

To visualize AT11-L2 under different conditions, a non-denaturing polyacrylamide gel (15%) electrophoresis was used. Sucrose (Sigma-Aldrich, USA) was added to the different samples at a concentration of 19%. To evaluate the molecularity of the G4 with the tested ligands, samples were prepared in a 1:2 ratio (AT11-L2/ligand) and incubated for 30 min. To test the influence of ligands on the G4/NCL complex, 2 µM of NCL was added to the sample and incubated for 30 min. Electrophoresis was performed at 120 V at room temperature. After electrophoresis, the gel was stained with SYBR gold (ThermoFisher Scientific, MA, USA) for 10 min with gentle agitation, and analyzed using ChemiDOC^TM^ XRS system (BioRad, Hercules, CA, USA).

### 3.8. Confocal Fluorescence Microscopy

Tongue cancer cell line UPCI-SCC-154 was grown in EMEM medium, containing 10% (*v*/*v*) FBS and 1% (*v*/*v*) penicillin-streptomycin. The cells were seeded at a density of 12.5 × 10^4^ cells per mL (200 μL in each well) in a treated μ-slide 8 well (ibidi, Germany) and grown at 37 °C in a 5% CO_2_ humidified atmosphere.

For NCL immunocytochemistry, the cells were incubated with the primary anti-NCL polyclonal antibody (PA3-16875, Invitrogen, Waltham, MA, USA) at 1:100 for 2 h at 37 °C, and then with a secondary antibody (Alexa Fluor 647^®^, A21244, Invitrogen, USA; dilution of 1: 1000) for 1 h at 37 °C. The cells were then stained with 2 μM of Hoechst 33342^®^ (h3570, Invitrogen™ Thermo Fisher Scientific, MA, USA) during 15 min. After that, cells were treated with 5 µM of pre-annealed AT11-L2 G4, which was labeled with 5′-FAM and 3′-TAMRA, and with 10 µM of PhenDC3 for 2 h at 37 °C. Between each step, the sample was washed off by rinsing with PBS three times.

The cells were observed in a Zeiss LSM 710 confocal laser scanning microscope (Zeiss, Germany) coupled with a plane-apochromat, and a 63×/DIC M27 objective was used to capture the fluorescence images. After image acquisition, the degree of AT11-L2 with PhenDC3 colocalization with NCL was expressed in Manders coefficient values, which were calculated using the JaCop tool in the FIJI software [27]. Colocalization coefficients were calculated from four images and Costes’ automatic threshold was used.

## 4. Conclusions

The AT11-L2 sequence, which was derived from AT11 by adding a single internal thymine, adopted a G4 hybrid topology in buffer containing low KCl concentrations and switched to a parallel G4 topology in the presence of 100 mM of KCl. Upon titration with ligands 360A, BRACO-19, PhenDC3 and PDS, the parallel G4 topology was maintained; however, in the presence of TMPyP4, no changes in pre-folded G4 hybrid topology were observed, and while adding more TMPyP4 promoted the disruption of G4, as previously reported for this ligand at high concentrations [28]. The thermal stability of AT11-L2 alone is modest but significantly improved in the presence of various G4 ligands. The *K*_*D*_ values showed that the interaction of the ligands with AT11-L2 is characterized by a medium-high affinity (10^−6^ M) and high affinity with NCL (10^−9^ M), suggesting that the ligands did not impair the binding to NCL. Additionally, AT11-L2 G4 colocalized with NCL at the cell surface of UPCI-SCC-154 cells, as suggested by confocal experiments using an anti-NCL antibody.

## Figures and Tables

**Table 1:** Thermal stabilization induced by ligands in the AT11-L2 sequence measured by CD melting experiments.

Ligand	Δ*T_m_* (°C) *				
	0.5 Eq.	1.0 Eq.	2.0 Eq.	3.5 Eq.	5.0 Eq.
360A	0.3	1.4	6.5	28.8	>30.0
BRACO-19	1.9	6.8	22.5	>30.0	>30.0
PhenDC3	1.0	3.2	14.9	>30.0	>30.0
TMPyP4	5.3	19.5	22.9	*	*
PDS	NS	NS	1.8	3.3	4.8

* Δ*T*_m_ represents the difference in melting temperature [Δ*T*_m_ = *T*_m_ (AT11-L2 + ligand) − *T*_m_ (AT11-L2)]. The experiments were performed in buffer containing 10 mM of lithium cacodylate (pH = 7.2) and 100 mM of KCl at 10 µM of AT11-L2. The *T*_m_ value for AT11-L2 alone is 38.9 °C. * structure is largely unfolded, as shown by Figure 3D. Negative Δ*T*_m_ values are reported as NS (no stabilization).

**Table 2:** Apparent dissociation constants (*K*_D_) of pre-folded 5′-Cy5-AT11-L2 G4 in the presence of the tested ligands obtained by fluorescence titrations.

Ligand	*K*_D_ (M)	*n*
360A	3.1 × 10^−5^ ± 6.0 × 10^−5^	0.6
TMPyP4	1.3 × 10^−6^ ± 1.5 × 10^−7^	1.2
PhenDC3	1.4 × 10^−6^ ± 8.5 × 10^−8^	2.2
BRACO-19	5.6 × 10^−6^ ± 2.0 × 10^−6^	0.9
PDS	1.4 × 10^−8^ ± 4.9 × 10^−9^	*

* Saturation binding plot fitted to Michaelis–Menten model.

## Data Availability

Not applicable.

## Supplementary Materials

The following are available online at https://www.mdpi.com/article/10.3390/molecules27206781/s1, Figure S1: Effect of temperature variation on AT11-L2 G4 monitored by 1H NMR spectroscopy in annealing buffer containing10 mM LiCaco (pH 7.2) supplemented with 100 mM KCl. Figure S2. Study of interaction among AT11-L2 G4 and PhenDC3 using 1H NMR spectroscopy. The spectra were recorded at 20 °C in 10 mM LiCaco supplemented with 100 mM of KCl, 8% DMSO-d6 and 10% D2O. Figure S3. 1H NMR temperature variation experiment of AT11-L2 G4/PhenDC3 complex at 37 °C. Figure S4-Fluorescence titration spectra of pre-folded 5′-Cy5-AT11-L2 sequence complexed with (A) 360A, (B) BRACO-19, (C) PhenDC3, (D) TMPyP4 and (E) PDS with increase concentrations of NCL. Figure S5. Predicted structure of AT11-L2 G4, obtained from the final snapshot of the 200 ns MD simulation. Nucleotides are highlighted in orange and the backbone is coloured in light grey. (A) Side view and (B) Top view. (C) RMSF plot of each nucleotide in the AT11-L2 G4 structure during the full 200 ns MD simulation. (D) RMSD plot of the 200 ns simulation of AT11-L2 G4 structure. Figure S6. Final snapshots (side and top view) and RMSD plots of 200 ns MD simulations of the complexes between AT11-L2 G4 and A) BRACO-19, B) 360A, C) PDS and D) TMPyP4. The backbone is highlighted in light grey, while nucleotides are depicted in orange. Potassium ions are shown in purple. The ligand is depicted in blue. Hydrogen bonds are depicted as dashed red lines. The first and last nucleotides of AT11-L2 are also shown. Table S1-NCL and 5´-FAM-AT11-L2-3´-TAMRA/PhenDC3 colocalization coefficients represented in mean ± SD.
