# Supplementary material for: G-Quadruplex Aptamer-Ligand Characterization
Source: Molecules. 2022 Oct 11;27(20):6781. doi: 10.3390/molecules27206781 (PMC9609330; doi:10.3390/molecules27206781)
Supplement: Supplementary file 1 [file molecules-27-06781-s001.zip › molecules-1922525-supplementary.pdf]

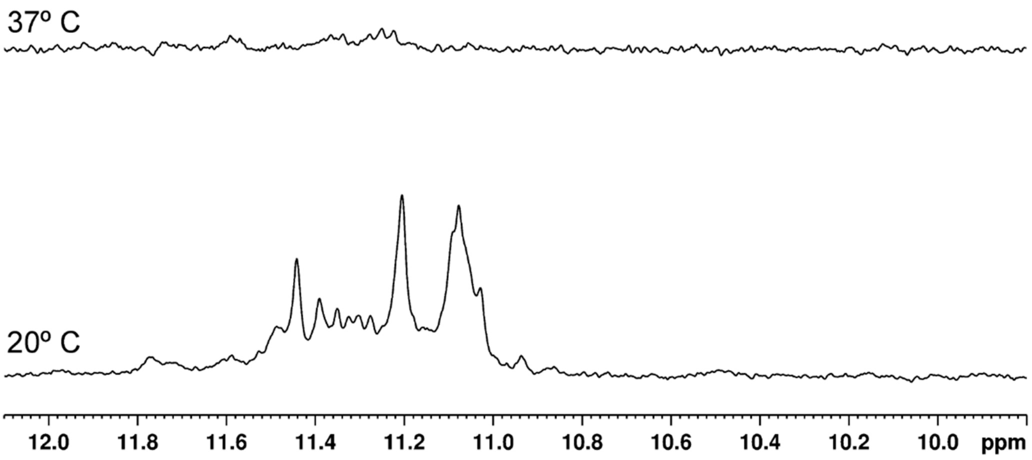

Figure S1. Effect of temperature variation on AT11-L2 G4 monitored by ^{1}H NMR spectroscopy in annealing buffer containing 10 mM lithium cacodylate (pH 7.2) supplemented with 100 mM KCl.

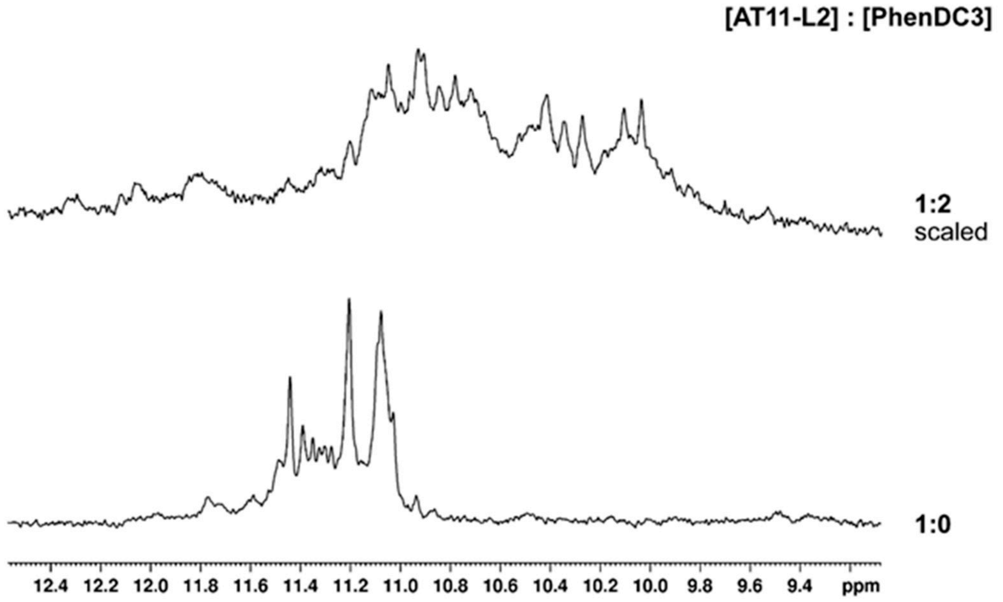

Figure S2. Study of interaction among AT11-L2 G4 and PhenDC3 using ^{1}H NMR spectroscopy. The spectra were recorded at 20ºC in 10 mM lithium cacodylate (pH 7.2) supplemented with 100 mM of KCl, 8% DMSO-d_6 and 10% D_2O.

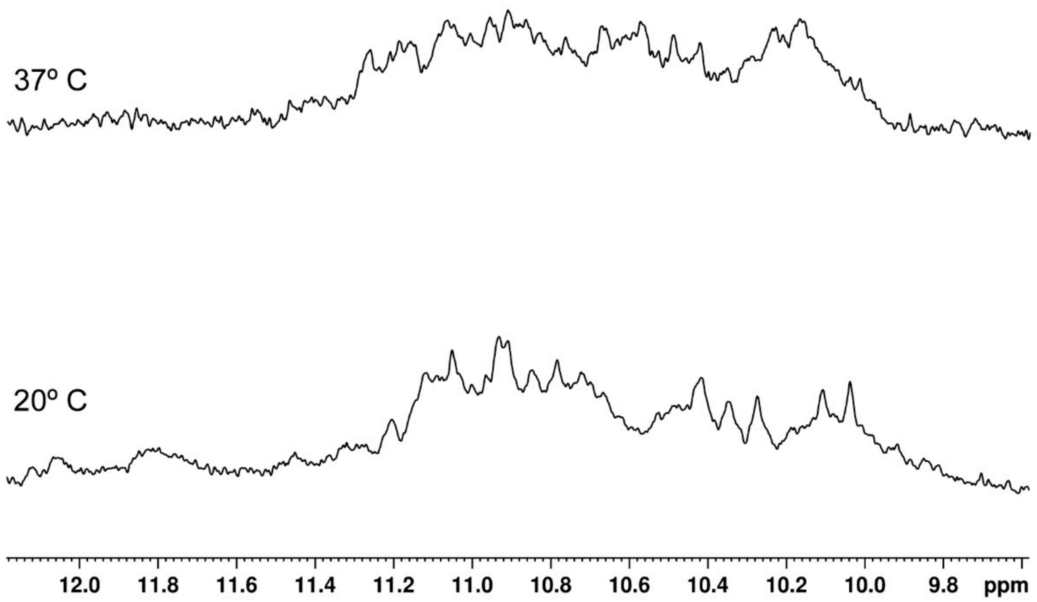

Figure S3. ^{1}H NMR temperature variation experiment of AT11-L2 G4/PhenDC3 complex at 37ºC.

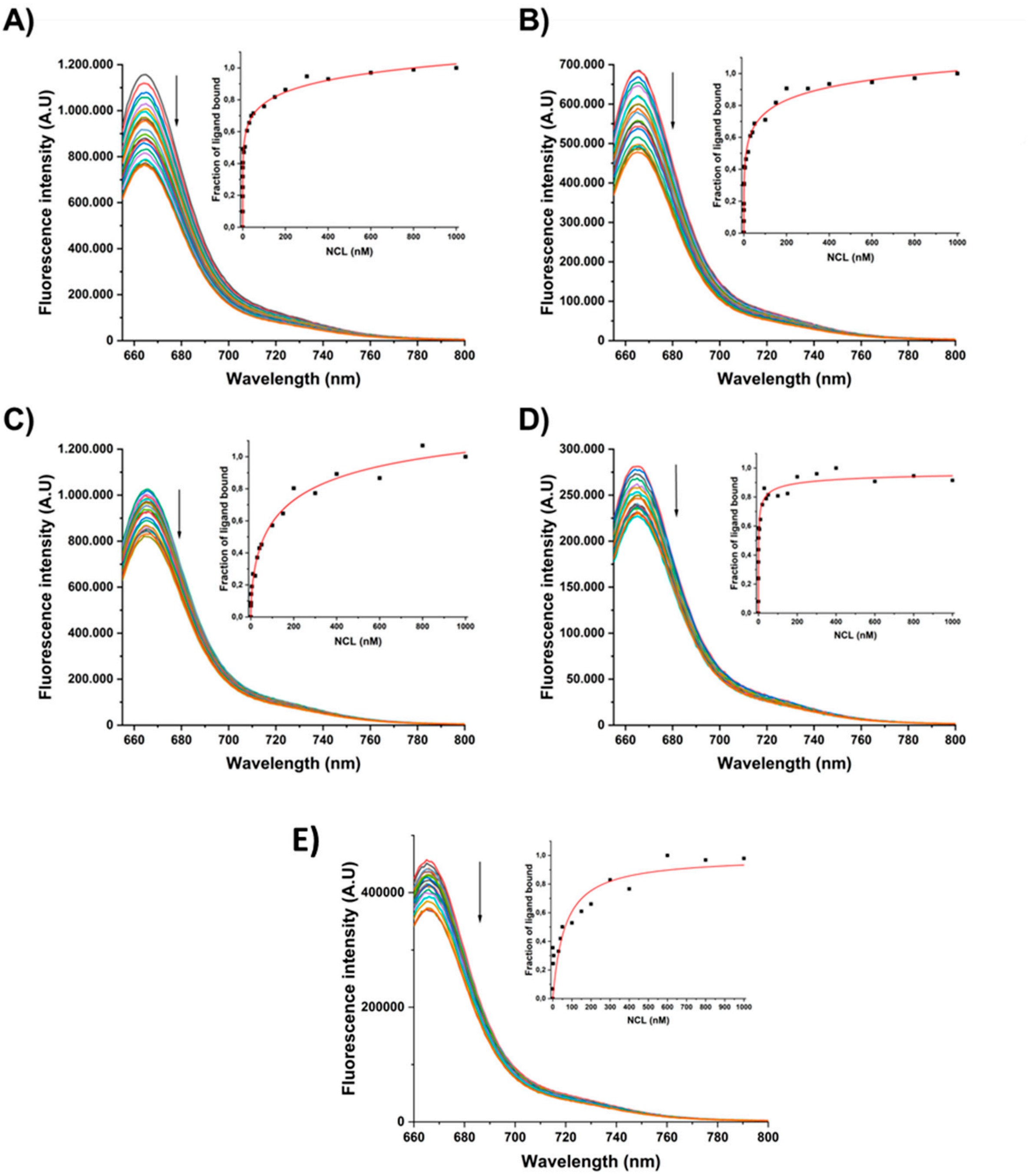

Figure S4. Fluorescence titration spectra of pre-folded 5'-Cy5-AT11-L2 sequence complexed with (A) 360A, (B) BRACO-19, (C) PhenDC3, (D) TMPyP4 and (E) PDS with increase concentrations of NCL.

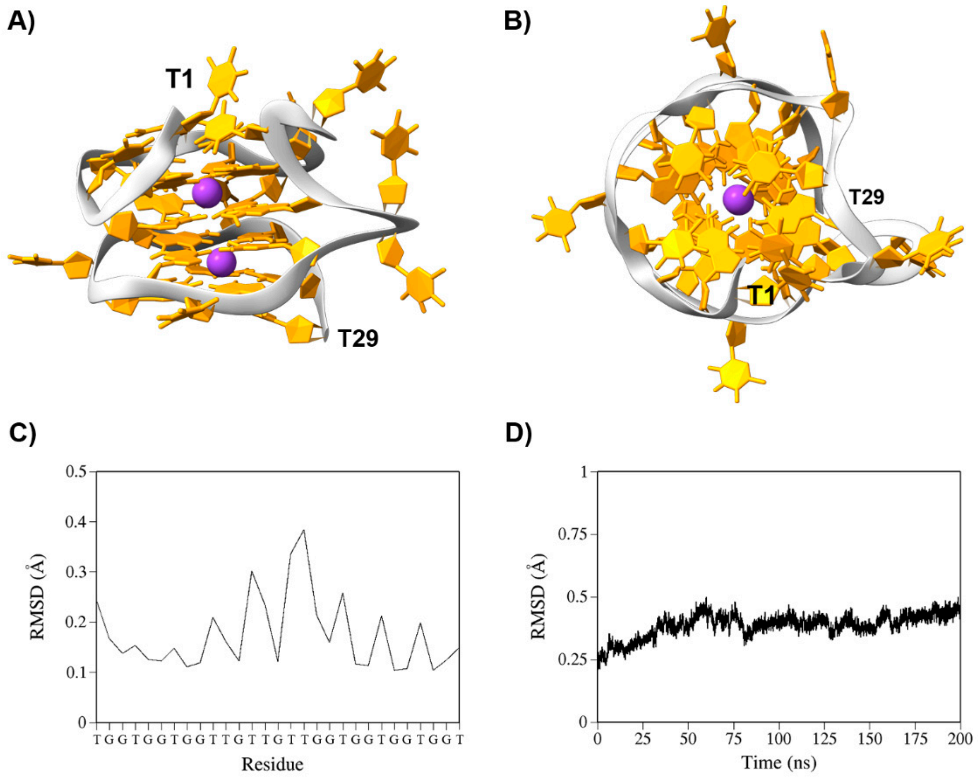

Figure S5. Predicted structure of AT11-L2 G4, obtained from the final snapshot of the 200 ns MD simulation. Nucleotides are highlighted in orange and the backbone is coloured in light grey. (A) Side view and (B) Top view. (C) RMSF plot of each nucleotide in the AT11-L2 G4 structure during the full 200 ns MD simulation. (D) RMSD plot of the 200 ns simulation of AT11-L2 G4 structure.

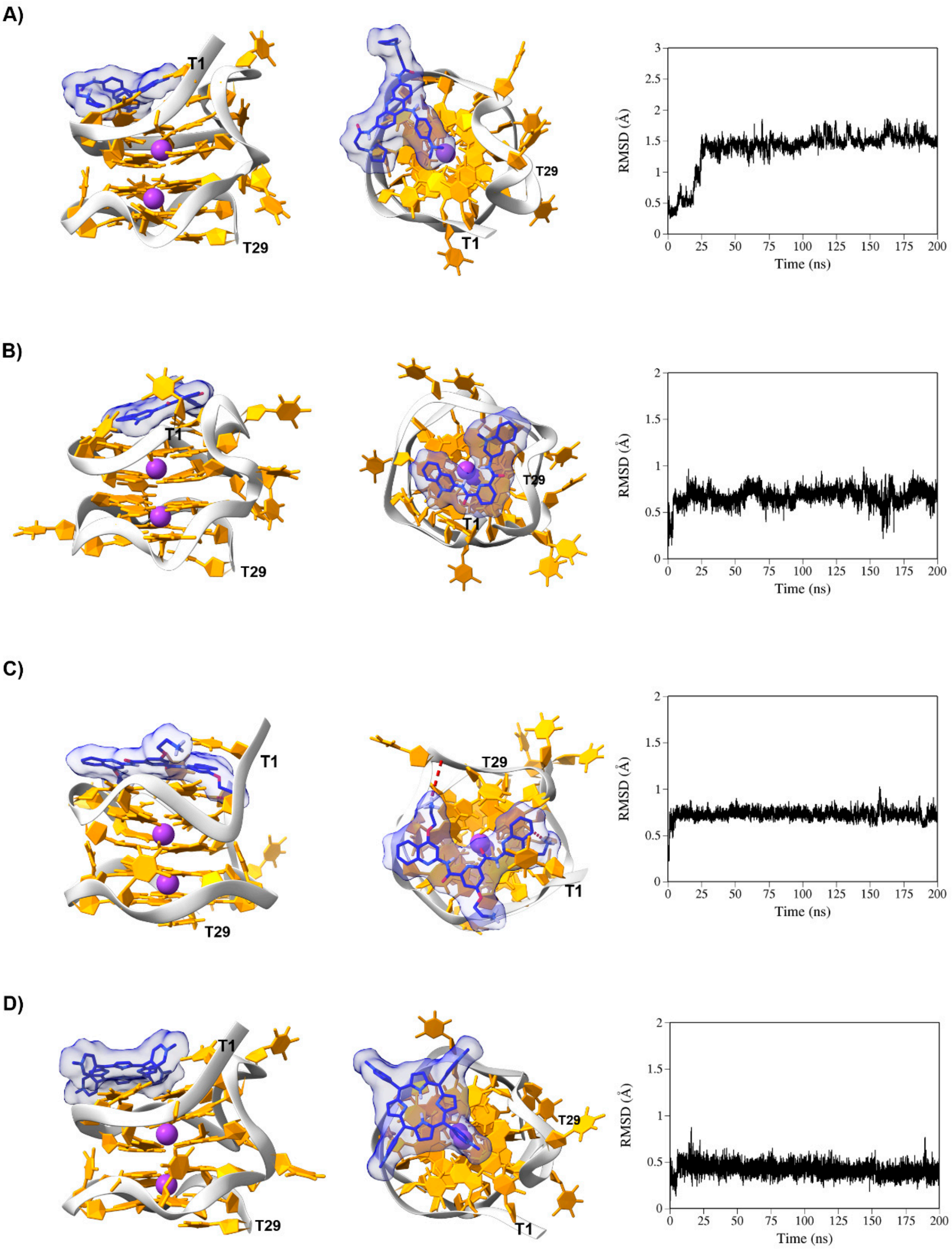

Figure S6. Final snapshots (side and top view) and RMSD plots of 200 ns MD simulations of the complexes between AT11-L2 G4 and A) BRACO-19, B) 360A, C) PDS and D) TMPyP4. The backbone is highlighted in light grey, while nucleotides are depicted in orange. Potassium ions are shown in purple. The ligand is depicted in blue. Hydrogen bonds are depicted as dashed red lines. The first and last nucleotides of AT11-L2 are also shown.

Table S1. NCL and 5´-FAM-AT11-L2-3´-TAMRA/PhenDC3 colocalization coefficients represented in mean ± SD.

**Coefficient**	**Value**
Manders' coefficient	$M_1 = 0.732 \pm 0.081$
	$M_2 = 0.733 \pm 0.030$

M_1 represents fraction of NCL overlapping 5´-FAM-AT11-L2-3´-TAMRA/PhenDC3 staining. M2 represents fraction of 5´-FAM-AT11-L2-3´-TAMRA/PhenDC3 overlapping NCL staining.
